# Dietary antioxidant intake and the risk of developing Barrett’s oesophagus and oesophageal adenocarcinoma

**DOI:** 10.1038/s41416-018-0113-y

**Published:** 2018-05-21

**Authors:** James H-E Kang, Robert Luben, Leo Alexandre, Andrew R Hart

**Affiliations:** 1 Norfolk and Norwich University Hospital NHS Foundation Trust, Norwich, NR4 7UY UK; 2 Norwich Medical School, University of East Anglia, Norwich, NR4 7UQ UK; 3 Strangeways Research Laboratories, Wort’s Causeway, Cambridge, CB1 8RN UK

**Keywords:** Risk factors, Oncology

## Abstract

**Background:**

We investigated in a cohort study, for the first time using 7-day food diaries (7-DFDs), for age-dependent inverse associations with antioxidants, which have anti-carcinogenic properties, and development of Barrett’s oesophagus (BO) and oesophageal adenocarcinoma (OAC).

**Methods:**

A total of 24,068 well individuals completed 7-DFDs and donated blood. Vitamins C and E, carotenes, zinc and selenium intakes, and plasma vitamin C were measured. Participants were monitored for 15 years for BO and OAC. Hazard ratios (HRs) were estimated for: quintiles of intake and in participants younger and >=65 years at recruitment, the midpoint of BO peak prevalence.

**Results:**

A total of 197 participants developed BO and 74 OAC. There were no significant associations between antioxidants and BO or OAC in the whole cohort or if >65 years at recruitment. In participants <65 years, for BO, there was an inverse trend across plasma vitamin C quintiles (trend HR = 0.82; 95% CI = 0.71–0.96, *P* = 0.01), OAC for plasma vitamin C (trend HR = 0.58; 95% CI = 0.37–0.92, *P* = 0.02) and for dietary vitamins C and E (trend HR = 0.71 95% CI = 0.51–0.99, *P* = 0.04 and trend HR = 0.70; 95% CI = 0.51–0.96; *P* = 0.03).

**Conclusions:**

Data supports a role for dietary antioxidants prevent BO and OAC, perhaps at the earlier stages of carcinogenesis.

## Introduction

The aetiology of both Barrett’s oesophagus (BO) and oesophageal adenocarcinoma (OAC), and the exposures that influence the malignant transformation of BO, is not fully understood. The carcinogenic processes may involve oxidative stress, whereby electrons are removed from: DNA, cellular proteins and membrane lipids.^1,2^ Dietary antioxidants, including vitamins C and E, beta-carotene, selenium and zinc may inhibit oxidation and protect against BO and OAC. For BO, results from epidemiological studies investigating these dietary micronutrients are inconsistent.^3–7^ Conversely, for OAC observational work consistently documents inverse associations with high-dietary antioxidant intakes.^3,6–11^ A meta-analysis of eight case-control studies of OAC reported such associations for the highest vs lowest quartiles of vitamin C (OR = 0.49, 95% CI = 0.39–0.62), beta-carotene (OR = 0.46, 95% CI = 0.36–0.59) and vitamin E (OR = 0.80, 95% CI = 0.63–1.03).^9^ However, all included studies were case-control investigations prone to both recall and selection biases. Furthermore, nutritional intakes were measured with semi-quantitative food frequency questionnaires (FFQs), which are less accurate than seven-day food diaries (7-DFDs).

We conducted a prospective cohort study to estimate, with more precision than previous work by using food diaries, if there were inverse associations between dietary antioxidants and the development of BO and OAC in the same population. To suggest whether dietary antioxidants influence either the earlier, later or both stages of carcinogenesis, we investigated micronutrient intake and the subsequent development of OAC differentially above and below the age of 65 years at recruitment. This age is the midpoint of the age range 60–69 years, which is the peak prevalence of BO diagnoses^12^ when at least metaplasia, with or without dysplasia is present. Demonstrating inverse associations with higher antioxidant intakes may support population-based dietary interventions to prevent this highly aggressive cancer and justify randomised controlled trials of antioxidants in inhibiting the malignant progression of BO to OAC.

## Methods

The cohort comprised 24,068 individuals, aged 40–79 years, in the European Prospective Investigation of Cancer-Norfolk (EPIC-Norfolk) study, recruited between 1993 and 1997. At recruitment participants completed questionnaires on: demography, habitual diet and lifestyle including smoking. Participants attended a baseline health check, conducted by a nurse, who took non-fasting blood samples and anthropometric measurements. The nurse explained the 7-DFD, the first day that was a 24-h recall of previous day’s intake. Participants completed the remaining 6 days at home documenting their entire intake. This included: food types, portion sizes, brands, cooking methods and recipes in eight separate meal and snack times. The 7-DFDs were returned and nutritionists inputted the data into a computer program called DINER (Data Into Nutrients for Epidemiological Research).^13^ Nutritionists matched each diary entry to one of 11,000 food items and 55,000 portion sizes within DINER, which best described it. DINER facilitated translation of participant-reported food consumption into structured nutrient data. Each 7-DFD had an average of 220 individual food and drink items reported. From this process, the daily intakes of vitamins C and E, zinc, selenium and carotenes were calculated. Plasma vitamin C was measured as a marker of bioavailability and intake. The cohort was monitored up 30 June 2015 to identify participants who developed either incident BO or OAC. Case notes were reviewed by clinical gastroenterologists, and to be included cases needed both endoscopic and histological verification.

In the analysis, micronutrients intakes from 7-DFDs and plasma vitamin C concentrations were divided into quintiles. There were 23,624 non-cases from the cohort, who had had their 7-DFDs coded. Cox proportional hazards models estimated hazard ratios (HRs) for developing BO or OAC separately for quintiles of antioxidants and plasma vitamin C. Analyses were adjusted for covariates of recruitment age and gender, and in a second model additionally body mass index (BMI), smoking, alcohol, energy intake, vitamin supplements and educational level. Analyses were repeated for participants younger and older than 65 years at recruitment, the mean of the reported midpoint of age range of BO.^12,14^ We performed tests for linear trend across quintile categories of intake.

## Results

During follow-up, 197 participants were diagnosed with BO and 74 for OAC, with data from 23,624 non-cases available (Table 1). Metaplasia was classed as either: intestinal 69%, gastric 10%, mosaic 10% and not reported 11%. In total, 5% of participants had dysplasia and 7% subsequently developed OAC. In the multivariable analyses of participants in the whole cohort (91% completed all 7 days of the diary), there were no statistically significant associations between any quintile of dietary vitamin C, vitamin E, zinc, selenium or carotenes, and the risk of either BO or OAC, and no trends across any quintiles (Tables 2 and 3, data on zinc, selenium and carotenes not shown. Similarly, there were no such associations when these analyses were repeated for participants older than 65 years at recruitment. However, in participants younger than 65 years at recruitment who developed BO, statistically non-significant inverse associations were observed between all quintiles of both food diary assessed and plasma vitamin C (highest vs lowest quintile of dietary vitamin C HR = 0.57, 95% CI = 0.31–1.04, *P* = 0.07; highest vs lowest quintile of plasma vitamin C HR = 0.48, 95% CI = 0.23–1.01, *P* = 0.06), with a significant inverse trend across quintiles for plasma vitamin C and BO risk (trend HR = 0.82; 95% CI = 0.71–0.96; *P* = 0.01). There were no associations in this younger age group with any of the other dietary antioxidants. For OAC, participants recruited younger than 65 years, there were non-significant inverse associations for all quintiles of both dietary and plasma vitamin C (highest vs lowest quintile of dietary vitamin C HR = 0.36, 95% CI = 0.07–1.83, *P* = 0.22; highest vs lowest quintile of plasma vitamin C HR = 0.30, 95% CI = 0.06–1.45, *P* = 0.14), with significant inverse trends across quintiles for both dietary vitamin C intake (trend HR = 0.71; 95% CI = 0.51–0.99; *P* = 0.04) and plasma vitamin C (trend HR = 0.58; 95% CI = 0.37–0.92; *P* = 0.02). In this younger age group, there was a significant inverse association across quintiles of vitamin E intake and OAC (trend HR = 0.70; 95% CI = 0.51–0.96; *P* = 0.03), but no associations for quintiles or trends of either zinc, selenium or carotenes.

**Table 1:** Baseline characteristics of participants

	Non-cases (*n* = 23, 624)	BO cases (*n* = 197)	OAC cases (*n* = 74)
*Gender*			
Male (*n*, %)	10, 865 (46.0%)	140 (71.1%)**	60 (81.1%)**
Age at recruitment (years, median, range)	58.8 (39.5–79.1)	60.4 (40.1–76.1)	66.9 (46.7–76.3)**
Age at diagnosis (years, median, range)	—	67.4 (47.0–91.0)	73.0 (47.0–91.0)
Time from recruitment to diagnosis (years, median, range) BMI (kg/m^2^, mean, standard deviation)	—	13.1 (1.2–20.8)	6.2 (0.6–11.7)
Smoking status (*n*, %)	26.4 (3.9)	27.0 (3.5)*	27.1 (4.0)
Never smokers	10814 (46.2%)	64 (32.8%)**	20 (27.4%)**
Former smokers	9879 (42.2%)	106 (54.4%)**	43 (58.9%)*
Current smokers	2729 (11.7%)	25 (12.8%)	10 (13.7%)
Alcohol intake (units/wk, median, range)	3.5 (0.0–121.0)	5.5 (0.0–53.0)*	2.5 (0.0–44.0)
Energy intake (kcal, median, range)	1969.9 (632.7–5618.9)	2175.6 (826.9–4121.3)**	2094.4 (1053.6–3399.6)
Vitamin supplement use (yes, %)	9828 (41.6%)	79 (40.1%)	23 (31.1%)
*Education level, i.e. formal qualifications (n, %)*			
None	8615 (36.5%)	79 (40.1%)	32 (43.2%)
O-level or equivalent	2424 (10.3%)	15 (7.6%)	6 (8.1%)
A-level or equivalent	9500 (40.2%)	83 (42.1%)	27 (36.5%)
Higher degree	3069 (13.0%)	20 (10.2%)	9 (12.2%)
*Nutrient intake from food diaries*			
Vitamin C (mg/day, median, range)	76.4 (0–665.1)	68.8 (16.0–1152.4)	74.8 (6.3–175.4)
Vitamin E (mg/day, median, range)	9.5 (0.3–74.5)	10.2 (1.3–32.5)*	9.5 (3.1–24.4)

*P < 0.05, **P < 0.001 between cases and controls

**Table 2:** Associations between quintiles of dietary antioxidant intake or plasma vitamin C and risk of BO

Nutrient intake from food diary (median, range)	Non-cases (*n* = 23624)	BO cases (*n* = 197)			
	*n*	*n*	HR^1^ (95% CI)	HR^2^ (95% CI)	HR^3^ (95% CI)
*Vitamin C (mg/day)*					
Q1 (0– < 46.4)	4722	43	1.00	1.00	1.00
Q2 (46.4– < 65.8)	4720	44	1.01 (0.66–1.55)	0.83 (0.49–1.39)	1.64 (0.72–3.73)
Q3 (65.8– < 89.1)	4724	40	0.93 (0.60–1.45)	0.88 (0.53–1.47)	1.12 (0.46–2.70)
Q4 (89.1– < 123.3)	4730	34	0.81 (0.51–1.30)	0.73 (0.42–1.28)	1.12 (0.46–2.73)
Q5 (123.3–1152.4)	4728	36	0.79 (0.49–1.27)	0.57 (0.31–1.04)	1.53 (0.65–3.57)
*P*-value for trend across quintiles	—	—	0.18	0.07	0.66
*Vitamin E (mg/day)*					
Q1 (0.3– < 6.8)	4736	29	1.00	1.00	1.00
Q2 (6.8– < 8.6)	4733	31	0.93 (0.55–1.57)	1.09 (0.57–2.12)	0.68 (0.28–1.66)
Q3 (8.6– < 10.5)	4723	41	1.14 (0.69–1.87)	1.23 (0.65–2.29)	0.99 (0.43–2.28)
Q4 (10.5– < 13.3)	4721	43	1.18 (0.72–1.93)	0.89 (0.46–1.71)	1.89 (0.88–4.02)
Q5 (13.3–75.0)	4711	53	1.31 (0.80–2.12)	1.28 (0.69–2.35)	1.28 (0.55–2.96)
*P*-value for trend across quintiles	—	—	0.10	0.56	0.10
*Plasma vitamin C (μmol/L)*					
Q1 (3.0– < 37.0)	4287	43	1.00	1.00	1.00
Q2 (37.0– < 49.5)	4037	43	1.07 (0.69–1.64)	0.84 (0.51–1.39)	2.01 (0.82–4.89)
Q3 (49.5– < 58.7)	4156	38	0.98 (0.62–1.54)	0.77 (0.45–1.32)	1.86 (0.75–4.60)
Q4 (58.7– < 69.1)	4363	24	0.72 (0.43–1.21)	0.50 (0.26–0.95)	1.67 (0.64–4.36)
Q5 (69.1–242.0)	3924	23	0.74 (0.42–1.31)	0.48 (0.23–1.01)	1.82 (0.66–4.99)
*P*-value for trend across quintiles	—	—	0.15	0.01	0.37

HR^1^, HR^2^ and HR^3^ adjusted for gender, recruitment age, smoking status, BMI, alcohol, energy intake (kcal), vitamin supplement usage and educational level (no formal qualifications/O-level/A-level/higher degree or equivalents) in: ^1^whole cohort, ^2^ participants younger and ^3^older than 65 years old at recruitment, respectively

**Table 3:** Associations between quintiles of dietary antioxidants and risk of OAC

Nutrient intake from food diary (min-max)	Non-cases (*n* = 23624)	OAC cases (*n* = 73)			
	*n*	*n*	HR^1^ (95% CI)	HR^2^ (95% CI)	HR^3^ (95% CI)
*Vitamin C (mg/day)*					
Q1 (0– < 46.4)	4722	12	1.00	1.00	1.00
Q2 (46.4– < 65.8)	4720	20	2.02 (0.94–4.32)	1.43 (0.50–4.06)	2.85 (0.90–8.97)
Q3 (65.8– < 89.1)	4724	14	1.50 (0.66–3.45)	0.49 (0.12–2.00)	3.09 (0.97–9.77)
Q4 (89.1– < 123.3)	4730	13	1.35 (0.58–3.18)	0.35 (0.07–1.78)	2.82 (0.87–9.12)
Q5 (123.3–1152.4)	4728	14	1.64 (0.72–3.75)	0.36 (0.07–1.83)	3.60 (1.14–11.24)
*P*-value for trend across quintiles	—	—	0.57	0.04	0.06
*Vitamin E (mg/day)*					
Q1 (0.3– < 6.8)	4736	15	1.00	1.00	1.00
Q2 (6.8– < 8.6)	4733	14	0.92 (0.44–1.91)	0.33 (0.09–1.24)	1.63 (0.63–4.23)
Q3 (8.6– < 10.5)	4723	18	1.00 (0.49–2.05)	0.39 (0.11–1.32)	1.70 (0.66–4.35)
Q4 (10.5– < 13.3)	4721	13	0.79 (0.37–1.70)	0.32 (0.09–1.13)	1.38 (0.51–3.78)
Q5 (13.3–75.0)	4711	13	0.67 (0.30–1.48)	0.20 (0.49–0.80)	1.35 (0.49–3.72)
*P*-value for trend across quintiles	—	—	0.10	0.03	0.80
*Plasma vitamin C (μmol/L)*					
Q1 (3.0– < 37.0)	4287	19	1.00	1.00	1.00
Q2 (37.0– < 49.5)	4037	13	0.89 (0.42–1.86)	0.38 (0.10–1.39)	1.54 (0.59–4.03)
Q3 (49.5– < 58.7)	4156	20	1.62 (0.84–3.14)	0.42 (0.11–1.59)	3.16 (1.33–7.49)
Q4 (58.7– < 69.1)	4363	6	0.61 (0.24–1.57)	0.30 (0.06–1.45)	0.99 (0.29–3.35)
Q5 (69.1–242.0)	3924	6	0.85 (0.32–2.25)	(no cases)	2.08 (0.69–6.27)
*P*-value for trend across quintiles	—	—	0.59	0.02	0.25

HR^1^, HR^2^ and HR^3^ adjusted for gender, recruitment age, smoking status, BMI, alcohol, energy intake (kcal), vitamin supplement usage and educational level (no formal qualifications/O-level/A-level/higher degree or equivalents) in: ^1^whole cohort, ^2^participants younger and ^3^older than 65 years old at recruitment, respectively

## Discussion

The main findings of this observational study were large inverse associations between both dietary and plasma vitamin C, dietary vitamin E and the risk of OAC, plus plasma vitamin C and BO, in participants at recruitment aged younger, but not older than, 65 years. Some evidence these associations may be protective ones are: plausible biological mechanisms for antioxidants preventing BO and OAC, large effect sizes, a biological gradient, associations persisting after correcting for confounders and temporality of dietary data collection, although to infer causality the findings need to be replicated in similar aetiological studies. The reasons for the inverse association between vitamins C and E and OAC in participants recruited younger than 65 years, the midpoint of the peak prevalence of symptomatically diagnosed BO, but not those older than 65 years, are uncertain. The molecular mechanisms for OAC involve metaplasia, dysplasia and malignant change. Our findings of inverse associations in participants recruited before the peak prevalence of BO and then the subsequent development of OAC is consistent with the hypothesis that pro-oxidation is involved in the earlier, rather than later histological changes in the oesophageal mucosa, which may be attenuated by dietary antioxidants. This epidemiological finding would support any laboratory mechanistic information showing earlier stages of carcinogenesis involve pro-oxidation.

The study’s strengths include its prospective design, which ensured that antioxidant intakes were assessed prior to symptoms, thereby reducing recall bias. A strength was the accuracy of the 7-day food diaries for measuring habitual dietary intake, which were validated against 16 day weighed records, the gold standard for dietary studies. For vitamin C intake, the Spearman correlation coefficient for 7-DFDs was 0.70 compared with 0.54 from FFQs,^15^ hence the former attenuate measurement error for diet. Follow-up bias should be minimal, as 20 years after EPIC recruitment, 95.6% of the population still have Norfolk post codes. In any observational study there is always the possibility of residual confounding, namely other dietary variables associated with antioxidant intake, which truly influence disease risk. There were relatively small numbers of participants in quintiles of antioxidants, although further follow-up will accrue more cases to give greater statistical precision. We acknowledge that inverse associations for antioxidants and OAC in the younger age group may be due to chance, although full statistical significance was reported for vitamins C and E. Only one diary record and plasma sample were recorded at baseline, and some individuals’ diets will alter due to illness and seasonal changes. However, as this is a prospective cohort design and measurement error is applicable to future cases and non-cases, effect sizes will be an under-estimate rather than spurious overestimates. Repeated measures of diet over time in a cohort study reported intake remained stable and was unlikely to change between quintile categories.^16^

No previous prospective cohort study, whose methodology reduces recall and selection biases, has investigated dietary antioxidants and the risk of developing both BO and OAC in the same population. Prospective data from our investigation suggests the inverse associations with certain antioxidants are more likely to be true ones, although confirmation from other cohort studies is required. Such data may support randomised controlled trials assessing if these micronutrients prevent the transformation of BO.
